# Cefazolin Inoculum Effect and Cefazolin Microbiological Treatment Failure in Serious Methicillin-Susceptible *Staphylococcus aureus* Infections: A Multicenter Retrospective Cohort Study

**DOI:** 10.1093/infdis/jiag199

**Published:** 2026-04-02

**Authors:** Mitchell A Jeffs, Nicole Li, Oluwalade Ogunkoya, D Brody Duncan, Carson K L Lo, Clayton W Hall, Ameera Nemer, Elaf Alzarnougi, Calvin Ka-Fung Lo, Emma B Monti, Thuwiba Al Baluki, Zong Heng Shi, Xena X Li, Prameet M Sheth, Christopher T Lohans, Anthony D Bai

**Affiliations:** Department of Biomedical and Molecular Sciences, Queen’s University, Kingston, Ontario, Canada; Department of Biomedical and Molecular Sciences, Queen’s University, Kingston, Ontario, Canada; Department of Biomedical and Molecular Sciences, Queen’s University, Kingston, Ontario, Canada; Department of Pathology and Molecular Medicine, Faculty of Health Sciences, McMaster University, Hamilton, Ontario, Canada; Hamilton Regional Laboratory Medicine Program, Hamilton Health Sciences, Hamilton, Ontario, Canada; Division of Infectious Diseases, Department of Medicine, McMaster University, Hamilton, Ontario, Canada; Division of Infectious Diseases, Department of Medicine, McMaster University, Hamilton, Ontario, Canada; Department of Pathology and Molecular Medicine, Faculty of Health Sciences, McMaster University, Hamilton, Ontario, Canada; Division of Infectious Diseases, Department of Medicine, McMaster University, Hamilton, Ontario, Canada; Division of Infectious Diseases, Department of Medicine, McMaster University, Hamilton, Ontario, Canada; Department of Pathology and Laboratory Medicine, University of British Columbia, Vancouver, British Columbia, Canada; School of Medicine, Queen’s University, Kingston, Ontario, Canada; Division of Infectious Diseases, Department of Medicine, Queen’s University, Kingston, Ontario, Canada; Department of Pharmacy, North York General Hospital, Toronto, Ontario, Canada; Shared Hospital Laboratory, Toronto, Ontario, Canada; Department of Microbiology, Sunnybrook Health Sciences Centre, Toronto, Ontario, Canada; Division of Infectious Diseases, Department of Medicine, North York General Hospital, Toronto, Ontario, Canada; Department of Pathology and Molecular Medicine, Queen's University, Kingston, Ontario, Canada; Department of Biomedical and Molecular Sciences, Queen’s University, Kingston, Ontario, Canada; Division of Infectious Diseases, Department of Medicine, Queen’s University, Kingston, Ontario, Canada

**Keywords:** cefazolin inoculum effect, *Staphylococcus aureus*, antibiotic treatment

## Abstract

**Background:**

It remains unclear if cefazolin inoculum effect (CzIE) translates to poorer clinical outcomes in patients with methicillin-susceptible *Staphylococcus aureus* (MSSA) infections who were treated with cefazolin.

**Methods:**

This retrospective cohort study across 5 hospitals in Ontario, Canada, from 2021 to 2025 included adult patients who received cefazolin treatment for serious MSSA infection based on blood or deep sterile site culture growth. CzIE was defined as a ≥4-fold increase in cefazolin minimum inhibitory concentration to ≥16 μg/mL at a high inoculum based on broth microdilution assay. Patients were followed to 90 days. Primary outcome was all-cause mortality. Secondary outcome was microbiological treatment failure based on culture growth of MSSA after 1 week of cefazolin treatment. Potential confounders were adjusted using propensity score weighting, and a competing risk model for microbiological treatment failure was used to account for mortality as a competing event.

**Results:**

Of 259 patients, 92 (35.5%) patients had an MSSA isolate that displayed CzIE. The 90-day mortality rate was 19/92 (20.7%) and 37/167 (22.2%) in the CzIE-positive and -negative groups, respectively, with adjusted risk difference of 0.6% (95% CI, −9.0% to 10.2%; *P* = .9060). Microbiological treatment failure occurred in 19/92 (20.7%) and 10/167 (6.0%) from the CzIE-positive and -negative groups, respectively, with adjusted subdistribution hazard ratio of 3.12 (95% CI, 1.38–7.08; *P* = .0065) in a competing risk model.

**Conclusions:**

CzIE was associated with a significantly increased risk of microbiological treatment failure. CzIE testing may be useful in guiding antibiotic treatment for serious MSSA infections.

## Teaser

In patients with serious methicillin-susceptible *Staphylococcus aureus* infections, cefazolin inoculum effect was associated with increased risk of microbiological treatment failure.

First-line antibiotics for treatment of methicillin-susceptible *Staphylococcus aureus* (MSSA) infections include cefazolin or an anti-staphylococcal penicillin such as cloxacillin [1, 2]. In a multicenter randomized controlled trial of patients with MSSA bacteremia, cefazolin was noninferior to cloxacillin with respect to clinical efficacy and had a significantly lower risk of acute kidney injury [3]. Therefore, cefazolin may be preferred over an anti-staphylococcal penicillin based on similar effectiveness and better safety.

However, there is concern about the effectiveness of cefazolin for MSSA infections with isolates displaying cefazolin inoculum effect (CzIE). Many definitions for CzIE exist [4–7]. Most commonly, CzIE is defined as a minimum inhibitory concentration (MIC) for cefazolin of ≤8 μg/mL at standard inoculum (∼5 × 10^5^ colony-forming units [CFU]/mL) that increases by ≥4 fold to ≥16 μg/mL into nonsusceptible range at high inoculum (∼5 × 10^7^ CFU/mL) by broth microdilution testing [4, 7]. CzIE is attributed to *blaZ* genes that encode type A and C β-lactamases [5]. In serious or deep-seated MSSA infections with large bacterial burden, there are theoretical concerns that the high production of such β-lactamases will hydrolyze and inactivate cefazolin, leading to cefazolin treatment failure [6].

It remains unclear if CzIE based on in vitro testing translates to cefazolin treatment failure for patients. In a systematic review of 23 observational studies, CzIE was not significantly associated with increased mortality risk in patients with MSSA infections treated with cefazolin [7]. However, the current evidence is limited by studies with small sample sizes, most of which did not adjust for confounders [7].

We conducted a multicenter retrospective cohort study of patients with serious MSSA infections treated with cefazolin to determine the association of CzIE with microbiological treatment failure at 90 days.

## MATERIALS AND METHODS

We conducted a multicenter retrospective cohort study. The study was approved by the research ethics board at each study site (Queen′s Health Sciences Research Ethics Board 6039751, Hamilton Research Ethics Board 17500, North York General Hospital Research Ethics Board #2024-0420-1096).

### Patient Population

The study included consecutive adult patients admitted to 5 hospitals in Ontario, Canada (Kingston General Hospital, Hamilton General Hospital, Juravinski Cancer Centre, St. Joseph's Healthcare Hamilton, and North York General Hospital) from 1 October 2021 to 31 May 2025. Patients needed to satisfy all of the following criteria to be included in the study:

1. Age 18 years or older.
2. MSSA isolated from blood or deep sterile site culture (deep abscess, bone, joint fluid, bronchoalveolar wash, or pleural fluid). MSSA was determined based on susceptibility testing done at the local microbiology laboratory according to Clinical and Laboratory Standards Institute (CLSI) standards.
3. Definitive antibiotic treatment with cefazolin (definitive antibiotic was the antibiotic initiated after susceptibility results were reported and given for minimum of 7 days or until treatment failure requiring switch to another antibiotic or until death [8]).
4. Saved MSSA isolates have been successfully recovered and regrown in culture for CzIE testing.

Therefore, this study included patients with MSSA bacteremia, deep abscesses, septic arthritis, osteomyelitis, and/or pneumonia, which would be considered serious *S aureus* infections [9, 10]. These study patients with serious MSSA infections were treated with cefazolin as the definitive antibiotic therapy.

### CzIE Testing

Difco Brain Heart Infusion (BHI) broth and BBL cation-adjusted Mueller–Hinton broth (CAMHB) powder were purchased from Becton Dickinson (Franklin Lakes, NJ, USA). Cefazolin sodium salt was purchased from Glentham Life Sciences (Corsham, United Kingdom).

All MSSA isolates from study patients were sent to our research laboratory (Queen's University at Kingston, Ontario, Canada) for CzIE testing. Only the isolate from the first positive MSSA culture was tested. BHI agar plates without antibiotic were inoculated with frozen cell stocks using a sterile inoculation loop and grown at 37°C under normal atmosphere for 16–20 hours without shaking. Each strain was then re-streaked on fresh BHI agar plates in preparation for the CzIE standard inoculum testing and cultured overnight, as described above. CzIE was tested using broth microdilution assays, based on published protocols [4], and in accordance with CLSI guidelines [11]. For the standard inoculum testing, 2-fold serial dilutions of cefazolin were prepared in a 96-well microplate (Falcon, flat-bottom, transparent, untreated) with CAMHB, such that the final concentrations of cefazolin ranged from 64 μg/mL to 0.06 μg/mL and final volumes were 180 µL in each well. MSSA colonies were suspended in CAMHB to achieve a turbidity of 0.5 McFarland units, corresponding to an inoculum of approximately 10^8^ CFU/mL. This suspension was then diluted and 20 µL of the diluted suspension added to each well of the 96-well microplate to achieve a final cell density of approximately 5 × 10^5^ CFU/mL (standard inoculum) [5, 6, 12]. For the high-inoculum preparation, 10 mL of liquid BHI broth was inoculated with an isolated colony of each MSSA isolate from the first plate culture. The cultures were grown for 16–20 hours at 37°C, 100 rpm in a shaking incubator [4]. The following day, 2-fold serial dilutions of cefazolin were prepared in a 96-well microplate (Falcon, flat-bottom, transparent, untreated) with CAMHB, such that the final cefazolin concentrations ranged from 64 μg/mL to 0.06 μg/mL and final volumes were 160 µL in each well. The overnight MSSA culture was diluted 4-fold in CAMHB, and then 40 µL of this suspension was added to each well in the 96-well plate, corresponding to a total dilution of the overnight culture of 20× [4]. Microplates for both the standard and high inocula were then incubated for 24 hours at 37°C, and growth was examined. The MIC corresponded to the lowest concentration of antibiotic for which there was no visible growth and was determined by 2 independent technicians via visual inspection of the plates. In cases of discrepancy, the 2 technicians would reexamine and reach a consensus on MIC.

CzIE was defined as positive when the MIC was ≤8 μg/mL with a standard inoculum (5 × 10^5^ CFU/mL), with subsequent increase by ≥4-fold to an MIC of ≥16 μg/mL with a high inoculum (5 × 10^7^ CFU/mL), which corresponded to the clinical breakpoint for cefazolin nonsusceptibility [4, 5, 6, 12]. Thus, isolates were required to fulfill both the 4-fold or higher increase in MIC as well as MIC ≥16 μg/mL at high inoculum to be classified as CzIE positive. For quality control, the *S aureus* TX0117 isolate was used as a CzIE-positive control whereas *S aureus* ATCC 29213 (BlaZ type A producer, CzIE negative) and *S aureus* ATCC 25923 (BlaZ negative, CzIE negative) were used as CzIE-negative controls [4]. These quality control strains were tested in each broth microdilution assay. In the initial validation of the assay, CFU/mL were verified for these control isolates under the standard and high-inoculum conditions.

### Outcome

Patients were followed for 90 days from culture collection. The primary outcome was 90-day all-cause mortality. Secondary outcome was microbiological treatment failure within 90 days, which was defined as growth of MSSA from any sterile site culture after 1 week of definitive antibiotic therapy. Microbiological treatment failure has been used as an outcome in other studies for *S. aureus* infections [13]. Days to microbiological treatment failure were calculated based on time from first positive culture collection date to the last positive culture collection date within the 90 days of follow-up.

Microbiological treatment failure would include both persistent infection (positive culture at <14 days from date of last positive culture of the first episode) and recurrent infection (positive culture occurring ≥14 days from date of last positive culture of the first episode) [14]. Intensity of follow-up culture was as per routine practice. In bacteremia, blood cultures were typically repeated every 2–3 days until clearance. Other sterile cultures were typically taken only in patients for whom there was clinically suspected persistent or recurrent infection.

### Data Collection

Patient data were collected retrospectively from the electronic medical record and entered into an online REDCap database. The clinician researcher who entered the data was blinded to the CzIE result. The following covariates at time of culture collection were collected:

1. Demographics: age, sex, and weight.
2. Comorbidities: Charlson Comorbidity Index [15], immunocompromised state including primary immunodeficiency, Human Immunodeficiency Virus HIV-1 infection, receipt of active chemotherapy, receipt of steroids (>10 mg of prednisone equivalent daily), neutropenia, recipient of a solid organ transplant or recipient of a stem cell transplant.
3. Acquisition: community acquired (positive culture within 48 hours of hospital admission) or nosocomial (positive culture after 48 hours of hospital admission).
4. Infectious foci: bacteremia, metastatic foci (≥2 infectious foci), line-associated bacteremia, infective endocarditis (including cardiac implantable electronic device related endocarditis), vascular graft infections, septic arthritis, prosthetic joint infection, osteomyelitis, skin and soft tissue infection, epidural abscess, other deep-seated abscesses, pneumonia, empyema, central nervous system infection, gastrointestinal tract infection, and urinary tract infection.
5. Illness severity: intensive care unit (ICU) admission and Pitt bacteremia score. The Pitt bacteremia score has been validated for both bacteremic and nonbacteremic infections [16, 17].

### Data Analysis

Patients were categorized into 2 groups: CzIE positive and CzIE negative. Baseline patient characteristics are described for each group. Descriptive analysis included mean ± standard deviation (SD) or median (interquartile range [IQR]) for continuous variables and counts (percentage) for categorical variables. Absolute standardized difference of the mean (ASDM) was used to describe the balance of baseline characteristics between the 2 groups. ASDM of >0.1 denotes a meaningful difference [18].

Overlap weighting using propensity scores was used to balance the measured covariates and estimate the average treatment effect for the overlap population [19, 20]. A logistic regression model was used to estimate the propensity score based on the following covariates: age, sex, hospital site, Charlson comorbidity index, hemodialysis, immunocompromised state, acquisition, infection foci, ICU admission, and Pitt bacteremia score. Generalized overlap weights were constructed as the product of the inverse probability weights and the harmonic mean of the generalized propensity scores [19]. Overlap weighting for 2 groups will always lead to the same proportion and mean between 2 groups as well as an ASDM of 0 for the covariates included in the propensity score [19, 21]. The weighted difference in means for the primary and secondary outcome yielded the average causal risk difference and its standard errors were used to estimate the 95% confidence interval (CI) [19].

For the secondary outcome of microbiological treatment failure, patients who died would not be at risk of future microbiological treatment failure. A competing risk model was used to account for death as a competing risk event. In this model, possible endpoints included microbiological treatment failure or death. The Fine and Gray model [22] was used to estimate subdistribution hazard ratio (sHR) for microbiological treatment failure. Overlap weights were entered into this model to estimate an adjusted sHR.

In a post hoc sensitivity analysis, follow-up was shortened from 90 days to 30 days to account for the possibility that attributable mortality and true recurrence with the same MSSA strain were more likely to occur in a shorter timeframe [23].

Three subgroup analyses were done. The first subgroup consisted of patients with bacteremia. The second subgroup consisted of patients with deep-seated infection at increased risk for high inoculum including infective endocarditis, vascular graft infection, septic arthritis, prosthetic joint infection, osteomyelitis, epidural abscess, other deep-seated abscess, and empyema. The third subgroup consisted of patients in whom source control was applicable stratified by if source control was achieved.

Statistical analysis was carried out using R version 4.2.3 (R Foundation for Statistical Computing). The PSweight package was used for overlap weighting of propensity scores [24].

## RESULTS

### Baseline Characteristics

**Figure 1.**
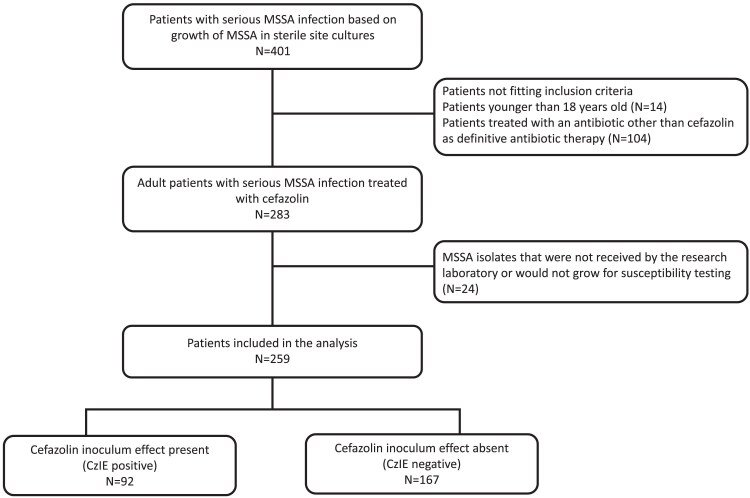
Flow diagram. Abbreviations: CzIE, cefazolin inoculum effect; MSSA, methicillin-susceptible *Staphylococcus aureus*.

Of 401 patients with serious MSSA infections, 259 patients were included in this study (Figure 1). Of the included patients, 92 (35.5%) patients had an MSSA isolate that displayed CzIE.

Baseline characteristics for the CzIE-positive and -negative groups are described in Table 1. Most patients had bacteremia (79.4% and 82.6% in the CzIE-positive and -negative groups, respectively). A significant proportion of patients had a deep-seated infection with potentially high inoculum (46.7% and 46.7% in the CzIE-positive and -negative groups, respectively).

**Table 1.** Baseline Characteristics

Characteristic	CzIE Positive (n = 92)	CzIE Negative (n = 167)	ASDM
Demographics			
Age, y, mean ± SD	66.6 ± 17.4	66.2 ± 16.4	0.0258
Sex			
Female	29 (31.5)	65 (38.9)	0.1554
Male	63 (68.5)	102 (61.1)	0.1554
Hospital site			
Hamilton General Hospital	5 (5.4)	17 (10.2)	0.1776
Juravinski Cancer Centre	5 (5.4)	15 (9.0)	0.1375
Kingston General Hospital	56 (60.9)	89 (53.3)	0.1535
North York General Hospital	23 (25.0)	36 (21.6)	0.0815
St. Joseph's Healthcare Hamilton	3 (3.3)	10 (6.0)	0.1301
Comorbidity			
Charlson comorbidity			
Myocardial infarction	11 (12.0)	18 (10.8)	0.0371
Congestive heart failure	10 (10.9)	24 (14.4)	0.1056
Peripheral vascular disease	5 (5.4)	10 (6.0)	0.0238
Cerebrovascular disease	3 (3.3)	22 (13.2)	0.3670
Dementia	9 (9.8)	10 (6.0)	0.1411
Hemiplegia	0 (0)	4 (2.4)	0.2215
Chronic pulmonary disease	10 (10.9)	27 (16.2)	0.1554
Connective tissue disease	3 (3.3)	6 (3.6)	0.0182
Peptic ulcer disease	3 (3.3)	5 (3.0)	0.0153
Mild liver disease	6 (6.5)	6 (3.6)	0.1340
Moderate to severe liver disease	4 (4.4)	8 (4.8)	0.0212
Chronic kidney disease	14 (15.2)	18 (10.8)	0.1323
Uncomplicated diabetes	20 (21.7)	29 (17.4)	0.1105
Complicated diabetes	17 (18.5)	33 (20.0)	0.0326
Localized solid tumor	5 (5.4)	13 (7.8)	0.0947
Metastatic solid tumor	3 (3.3)	9 (5.4)	0.1048
Leukemia or lymphoma	9 (9.8)	9 (5.4)	0.1665
HIV/AIDS	0 (0)	0 (0)	0
CCI score			
0	16 (17.4)	40 (24.0)	0.1625
1	25 (27.2)	35 (21.0)	0.1458
2	19 (20.7)	20 (12.0)	0.2364
≥3	32 (34.8)	72 (43.1)	0.1715
Hemodialysis	8 (8.7)	13 (7.8)	0.0331
Immunocompromised state	14 (15.2)	23 (13.8)	0.0411
Acquisition			
Community acquired	64 (69.6)	118 (70.7)	0.0239
Nosocomial	28 (30.4)	49 (29.3)	0.0239
Microbiology			
Polymicrobial growth	17 (18.5)	20 (12.0)	0.1817
Infection foci			
Bacteremia	73 (79.4)	138 (82.6)	0.0838
Metastatic foci	14 (15.2)	23 (13.8)	0.0411
Line-associated bacteremia	16 (17.4)	29 (17.4)	0.0007
Infective endocarditis	6 (6.5)	14 (8.4)	0.0709
Vascular graft infection	3 (3.3)	2 (1.2)	0.1401
Septic arthritis	9 (9.8)	11 (6.6)	0.1168
Prosthetic joint infection	9 (9.8)	21 (12.6)	0.0887
Osteomyelitis	10 (10.9)	23 (13.8)	0.0884
Skin and soft tissue infection	20 (21.7)	39 (23.4)	0.0386
Epidural abscess	8 (8.7)	10 (6.0)	0.1040
Other deep-seated abscess	8 (8.7)	17 (10.2)	0.0508
Pneumonia	7 (7.6)	13 (7.8)	0.0066
Empyema	0 (0)	2 (1.2)	0.1557
CNS infection	0 (0)	3 (1.8)	0.1913
Gastrointestinal tract infection	2 (2.2)	2 (1.2)	0.0759
Urinary tract infection	3 (3.3)	10 (6.0)	0.1301
Infection severity			
ICU admission within 48 h	19 (20.7)	27 (16.2)	0.1159
Pitt bacteremia score, mean ± SD	1.9 ± 2.5	1.8 ± 2.2	0.0457

Data represent No. (%) unless otherwise indicated.

Abbreviations: ASDM, absolute standardized difference of the mean; CCI, Charlson Comorbidity Index; CNS, central nervous system; CzIE, cefazolin inoculum effect; HIV, human immunodeficiency virus; ICU, intensive care unit; SD, standard deviation.

### Management of Infection

The mean ± SD days from culture collection to first dose of cefazolin was 1.8 ± 1.7 and 1.7 ± 1.4 days in the CzIE-positive and -negative groups, respectively. Cefazolin was initiated within 3 days from culture collection in 84 (91.3%) and 157 (94.0%) patients from the CzIE-positive and -negative groups, respectively. In patients with normal renal function, the most used cefazolin dose was 2 grams intravenously every 8 hours in 67/78 (85.9%) and 130/149 (87.2%) patients for the CzIE-positive and -negative groups, respectively. For context, the median (IQR) weight was 82.0 (71.0–100.0) kg in the CzIE-positive group and 78.0 (64.0–93.5) kg in the CzIE-negative group. The median (IQR) duration of cefazolin treatment was 26.5 (14.0–42.0) days and 27.0 (14.0–42.0) days in the CzIE-positive and -negative groups, respectively. On average, cefazolin treatment accounted for 89% and 84% of total antibiotic duration for the CzIE-positive and -negative groups, respectively.

In patients for whom source control such as line removal, device removal, or abscess drainage was applicable, source control was achieved in 47/57 (82.5%) and 89/103 (86.4%) patients in the CzIE-positive and -negative groups, respectively (Supplementary Table 1). Median (IQR) days from culture collection to source control was 1 (0–2) and 2 (1–4) in the CzIE-positive and -negative groups, respectively.

### Outcomes

**Figure 2.**
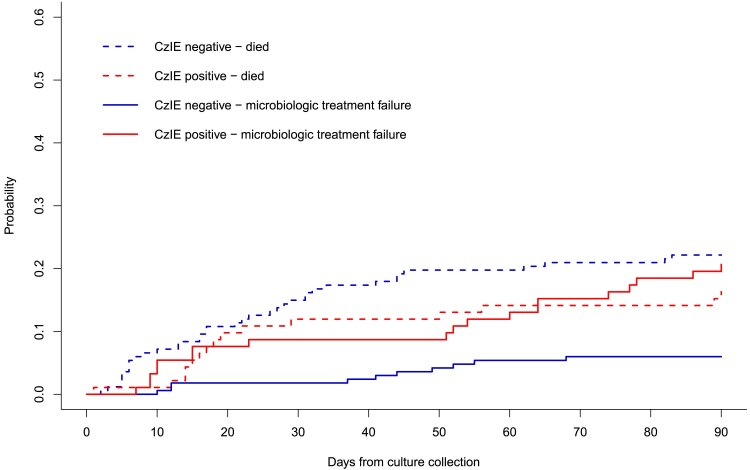
Cumulative incidence function of time to microbiological treatment failure or death in cefazolin inoculum effect (CzIE)–positive and –negative groups.

All patients were followed to day 90 with complete data for the primary and secondary outcome. Outcomes are described in Table 2. At 90 days, a similar proportion of patients died in the CzIE-positive and -negative groups (20.7% vs 22.2%, *P* = .8750) (Supplementary Table 2). However, a significantly higher proportion of patients in the CzIE-positive group had microbiological treatment failure (20.7% vs 6.0%, *P* = .0007) (Supplementary Table 3). Of the 19 patients who had microbiological treatment failure in the CzIE-positive group, 7 (36.8%) had persistent infection and 12 (63.2%) had recurrent infection. Of these 19 microbiological treatment failure cases, MSSA grew in culture from the same anatomic site in 16 (84.2%) cases. Of the 10 patients who had microbiological treatment failure in the CzIE-negative group, 3 (30.0%) had persistent infection and 7 (70.0%) had recurrent infection. Of these 10 microbiological treatment failure cases, MSSA grew in culture from the same anatomic site in 6 (60.0%) cases. The cumulative incidence function of time to microbiological treatment failure or death is shown in Figure 2. In a competing risk model, CzIE had an unadjusted sHR of 3.69 (95% CI, 1.72–7.94; *P* = .0008) for microbiological treatment failure.

**Table 2.** Outcomes Between Methicillin-Susceptible *Staphylococcus aureus* Infections With Cefazolin Inoculum Effect–Positive and –Negative Isolates

Outcome at 90 Days	CzIE Positive (n = 92)^a^	CzIE Negative (n = 167)^b^	Unadjusted Risk Difference (95% CI)	Adjusted Risk Difference (95% CI)
Mortality	19 (20.7)	37 (22.2)	−1.5% (−11.5% to 9.5%) *P* = .8750	0.6% (−9.0% to 10.2%) *P* = .9060
Microbiological treatment failure	19 (20.7)	10 (6.0)	14.7% (6.4%–24.6%) *P* = .0007	13.1% (4.8%–21.4%) *P* = .0021

Data represent No. (%) unless otherwise indicated.

Abbreviations: CI, confidence interval; CzIE, cefazolin inoculum effect.

^a^For the 92 patients in the CzIE-positive group at end of 90 days of follow-up: 58 patients were alive without microbiological treatment failure, 15 patients had microbiological treatment failure but survived, 15 patients did not have microbiological treatment failure but died, and 4 patients had microbiological treatment failure then died.

^b^For the 167 patients in the CzIE-negative group at end of 90 days of follow-up: 120 patients were alive without microbiological treatment failure, 10 patients had microbiological treatment failure but survived, 37 patients did not have microbiological treatment failure but died, and 0 patients had microbiological treatment failure then died.

### Adjustment for Covariates

The overlap population after weighting using propensity score is described in Table 3. The ASDM was 0 for all covariates used to estimate the propensity score as listed in Table 3 after overlap weighting.

**Table 3.** Baseline Characteristics After Overlap Weighting Using Propensity Scores

Characteristic	CzIE Positive	CzIE Negative
	Effective Sample Size = 85.7	Effective Sample Size = 133.6
Demographics		
Age, y, mean ± SD	66.7 ± 17.9	66.7 ± 17.0
Sex		
Female	32.8%	32.8%
Male	67.2%	67.2%
Hospital site		
Hamilton General Hospital	6.7%	6.7%
Juravinski Cancer Centre	6.5%	6.5%
Kingston General Hospital	58.3%	58.3%
North York General Hospital	24.4%	24.4%
St. Joseph's Healthcare Hamilton	4.1%	4.1%
Comorbidity		
CCI score		
0	19.9%	19.9%
1	26.8%	26.8%
2	16.1%	16.1%
≥3	37.2%	37.2%
Hemodialysis	7.5%	7.5%
Immunocompromised state	14.1%	14.1%
Acquisition		
Community acquired	68.6%	68.6%
Nosocomial	31.4%	31.4%
Microbiology		
Polymicrobial growth	16.5%	16.5%
Infection foci		
Bacteremia	80.6%	80.6%
Metastatic foci	14.6%	14.6%
Line-associated bacteremia	17.6%	17.6%
Infective endocarditis	6.7%	6.7%
Vascular graft infection	1.9%	1.9%
Septic arthritis	8.5%	8.5%
Prosthetic joint infection	11.4%	11.4%
Osteomyelitis	12.2%	12.2%
Skin and soft tissue infection	24.7%	24.7%
Epidural abscess	6.7%	6.7%
Other deep-seated abscess	9.2%	9.2%
Pneumonia	7.1%	7.1%
Empyema	0%	0%
CNS infection	0%	0%
Gastrointestinal tract infection	1.4%	1.4%
Urinary tract infection	3.9%	3.9%
Infection severity		
ICU admission within 48 h	19.9%	19.9%
Pitt bacteremia score, mean ± SD	2.0 ± 2.5	2.0 ± 2.3

Overlap weighting creates overlap population with same proportion and mean between 2 groups.

Abbreviations: CCI, Charlson Comorbidity Index; CNS, central nervous system; CzIE, cefazolin inoculum effect; ICU, intensive care unit; SD, standard deviation.

The adjusted risk differences after overlap weighting using propensity scores are described in Table 2. The mortality risk was similar between the 2 groups with adjusted risk difference of 0.6% (95% CI, −9.0% to 10.2%; *P* = .9060).

In a competing risk model after accounting for death, CzIE was associated with a significantly higher risk of microbiological treatment failure based on an adjusted sHR of 3.12 (95% CI, 1.38–7.08; *P* = .0065).

### Sensitivity Analysis

Sensitivity analysis restricting follow-up to 30 days showed similar results (Supplementary Table 4). CzIE was still associated with a significantly higher risk of microbiological treatment failure (adjusted sHR, 4.43 [95% CI, 1.03–19.1]; *P* = .0460).

### Subgroup Analyses

In the subgroup analyses, the microbiological treatment failure rate was consistently higher in the CzIE-positive group. In the MSSA bacteremia subgroup, microbiological treatment failure occurred in 13/73 (17.8%) and 10/138 (7.3%) in the CzIE-positive and -negative groups, respectively (Supplementary Table 5). In the subgroup of patients with deep-seated infection at risk for high inoculum, microbiological treatment failure occurred in 15/43 (34.9%) and 5/78 (6.4%) in the CzIE-positive and -negative groups, respectively (Supplementary Table 6). The CzIE-positive group had a higher proportion of microbiological treatment failure in patients for whom source control was applicable irrespective of whether source control was achieved or not (Supplementary Table 7).

## DISCUSSION

In this multicenter retrospective cohort study of 259 patients with serious MSSA infection treated with cefazolin, the CzIE prevalence was 35.5%. Although all-cause mortality was similar between the CzIE-positive and -negative groups, CzIE was associated with a higher risk of microbiological treatment failure with adjusted sHR of 3.12 (95% CI, 1.38–7.08; *P* = .0065).

Our study finding of CzIE presence not being significantly associated with mortality is consistent with prior studies. In a systematic review [7], CzIE was not significantly associated with mortality in the 2 observational studies that examined this association in patients [25, 26]. These 2 studies had small sample sizes that included only 10 and 13 patients in the CzIE-positive group treated with cefazolin [25, 26]. In contrast, our study had more than 4 times the number of patients in previous studies combined in the CzIE-positive group, allowing for a more precise estimate. In addition to the studies cited in the systematic review, 2 other observational studies demonstrated that CzIE was associated with increased mortality risk in MSSA bacteremia and endocarditis [27, 28]. However, our study patients differed greatly from these studies, because only 36% of study patients received cefazolin in one study [27] and 69% of study patients received a cephalosporin in the other study [28]. Regarding cefazolin treatment failure, the systematic review found 4 relevant observational studies [5, 6, 25, 26]. Of these studies, there was a trend of higher treatment failure in the CzIE-positive group, with odds ratios (ORs) ranging from 0.26 to 13.0 [7]. Only 1 study showed a significant association, with OR of 3.93 (95% CI, 1.08–14.3) [26]. Our study also reports a significant association between CzIE and microbiological treatment failure. Limitations of the previous 4 studies included small sample size ranging from 12 to 77 and no adjustment for potential confounders. In contrast, our study had more than 3 times the sample size of the largest study to date (N = 77) [25] and adjusted for potential confounders using propensity score. The definition of treatment failure varied across the prior studies [5, 6, 25, 26]. Our study used microbiological treatment failure, a more objective outcome that was proven by culture and captured both persistent and recurrent infection within 90 days.

Our study findings carry important clinical implications. The association between CzIE and microbiological treatment failure provides further evidence that CzIE testing may have a role in guiding antibiotic treatment decisions for serious MSSA infections. In patients with MSSA isolates displaying CzIE, cefazolin may be avoided to decrease risk of treatment failure.

Our study has many strengths. First, to our knowledge, this was the largest study to date on exploring the association between CzIE and clinical outcomes in patients with MSSA infection being treated with cefazolin. Second, we used a clinically relevant definition of CzIE based on an increase in MICs to above the clinical MIC breakpoint. Third, our study adjusted for prognostic factors using propensity score weighting. Fourth, a significant proportion of study patients had a deep-seated infection or metastatic foci in which CzIE would be more clinically relevant given the potential for higher inoculum size in those cases.

There are several limitations that merit mentioning. First, there are inherent limitations to any observational study where residual confounding is always a possibility. However, this study adjusted for many known prognostic factors including demographics, comorbidity, infectious foci, and illness severity using propensity score weighting. Second, the CzIE-positive group treated with cefazolin was not compared to an anti-staphylococcal penicillin because there were too few patients treated with an anti-staphylococcal penicillin at the study hospital sites. This reflected the local clinical practice of cefazolin being first-line treatment. The comparison to anti-staphylococcal penicillin would eliminate the possibility that MSSA isolates displaying CzIE may also possess other intrinsic bacterial characteristics that make the isolates more virulent, leading to treatment failure regardless of antibiotic choice [3, 29]. Third, we did not perform any sequence testing on the microbiological treatment failure cases to confirm that it was the same MSSA strain causing the recurrent infection. A different MSSA strain may have caused a new infection that was counted as microbiological treatment failure in the study. This possibility would be less likely when restricting the follow-up period to 30 days. Using this shorter 30-day timeframe in the sensitivity analysis, CzIE was still associated with an increased risk of microbiological treatment failure. Last, we did not perform molecular typing to determine *blaZ* type β-lactamases in isolates. It should be noted that *blaZ* genotype is only correlated with CzIE. For example, in a study of 641 genomes from blood culture MSSA isolates, type A *blaZ* gene was found in 78% of CzIE-positive isolates and 22% of CzIE-negative isolates [30].

Ideally, a randomized controlled trial of cefazolin versus anti-staphylococcal penicillin in patients with deep-seated infection due to MSSA isolates displaying CzIE would overcome the aforementioned limitations. We look forward to the publication of results from the *Staphylococcus aureus* Network Adaptive Platform (SNAP) trial [13] that is comparing cefazolin to anti-staphylococcal penicillin in MSSA bacteremia. One crucial subgroup analysis of the SNAP trial results would be reviewing microbiological treatment failure rates for cefazolin versus anti-staphylococcal penicillin in the deep-seated infection and CzIE-positive subgroup.

While waiting for this definitive trial, our study adds to the supporting evidence that CzIE may be clinically important given its association with an increased risk of cefazolin microbiological treatment failure in MSSA infections.

## Supplementary Material

jiag199_Supplementary_Data

## Supplementary Data

Supplementary materials are available at *The Journal of Infectious Diseases* online (http://jid.oxfordjournals.org/). Supplementary materials consist of data provided by the author that are published to benefit the reader. The posted materials are not copyedited. The contents of all supplementary data are the sole responsibility of the authors. Questions or messages regarding errors should be addressed to the author.
