# Supplementary material for: Cefazolin Inoculum Effect and Cefazolin Microbiological Treatment Failure in Serious Methicillin-Susceptible Staphylococcus aureus Infections: A Multicenter Retrospective Cohort Study
Source: J Infect Dis. 2026 Apr 2;233(6):e1433–42. doi: 10.1093/infdis/jiag199 (PMC13271370; doi:10.1093/infdis/jiag199)
Supplement: jiag199_Supplementary_Data [file jiag199_supplementary_data.docx]

Supplement Table 1. Description of type of source control

|  | CzIE positive | CzIE negative |
| --- | --- | --- |
| Line removal | 12/16 (75.0%) | 22/29 (75.9%) |
| Joint washout for septic arthritis | 9/9 (100%) | 7/11 (63.6%) |
| Joint surgery for prosthetic joint infection | 9/9 (100%) | 20/21 (95.2%) |
| Aspirate or drainage of deep abscesses | 7/8 (87.5%) | 11/17 (64.7%) |
| Spine surgery for epidural abscess | 7/8 (87.5%) | 7/10 (70.0%) |

Supplement Table 2. Time of death from culture collection date

| Group | Time of death from culture collection date in days |
| --- | --- |
| CzIE positive  (19 deaths) | 1, 12, 14, 14, 15, 16, 17, 17, 18, 19, 22, 29, 29, 29, 33, 50, 56, 89, 90  (1 patient died before completing 7 days of cefazolin) |
| CzIE negative  (37 deaths) | 2, 3, 5, 5, 5, 5, 6, 6, 6, 7, 8, 10, 13, 13, 16, 16, 17, 17, 21, 22, 23, 26, 27, 28, 29, 31, 31,  32, 34, 41, 44, 44, 45, 62, 65, 82, 83  (11 patients died before completing 7 days of cefazolin) |

Supplement Table 3. Description of microbiological treatment failure (N=29)

| Study ID | CzIE | Initial foci | Initial culture | Microbiologic treatment failure foci | Microbiologic treatment failure culture | 90-day outcome |
| --- | --- | --- | --- | --- | --- | --- |
| H079 | Positive | Line associated bacteremia | Blood | Recurrent bacteremia on day 54 | Blood | Survived |
| K097 | Positive | Prosthetic joint infection with bacteremia | Joint fluid  Blood | Persistent prosthetic joint infection with positive culture on day 15 | Joint fluid | Died |
| K099 | Positive | Skin and soft tissue infection with bacteremia | Blood | New septic arthritis on day 51 | Joint fluid | Survived |
| K105 | Positive | Bacteremia with epidural abscess | Blood  Abscess | Persistent bacteremia on day 9 | Blood | Survived |
| K109 | Positive | Prosthetic joint infection with bacteremia | Joint fluid  Blood | Persistent bacteremia and prosthetic joint infection on day 10 | Joint fluid  Blood | Survived |
| K226 | Positive | Prosthetic joint infection | Joint fluid | Recurrent prosthetic joint infection on day 74 | Joint fluid | Survived |
| K008 | Positive | Skin and soft tissue infection complicated by bacteremia with metastatic foci including septic arthritis, psoas abscess | Blood | New epidural abscess on day 86 | Epidural abscess | Survived |
| N025 | Positive | Infected hematoma with bacteremia and osteomyelitis | Blood | Persistent bacteremia on day 10 | Blood | Survived |
| N027 | Positive | Pneumonia with bacteremia | Blood  Sputum | Recurrent bacteremia on day 90 | Blood | Survived |
| N031 | Positive | Septic arthritis with bacteremia | Joint fluid  Blood | Persistent septic arthritis on washout on day 9 | Joint fluid | Survived |
| N079 | Positive | Line associated bacteremia, septic thrombophlebitis and infective endocarditis | Blood | Persistent bacteremia on day 7 | Blood | Died |
| K023 | Positive | Prosthetic joint infection and bacteremia | Blood | Persistent prosthetic joint infection on day 15 | Joint fluid | Died |
| K025 | Positive | Line associated bacteremia | Blood | Recurrent bacteremia on day 52 | Blood | Survived |
| K030 | Positive | Bacteremia with spine infection (osteomyelitis, epidural abscess and psoas abscess) | Fluid aspirate  Blood | Recurrent psoas abscess on day 78 | Fluid aspirate | Survived |
| K041 | Positive | Septic arthritis | Joint fluid | Recurrent septic arthritis on day 77 | Joint fluid | Survived |
| K054 | Positive | Prosthetic joint infection | Joint fluid | Recurrent prosthetic joint infection on day 64 | Joint fluid | Survived |
| K169 | Positive | Bacteremia and infective endocarditis | Blood | Recurrent bacteremia on day 60 | Blood | Survived |
| K261 | Positive | Vascular graft infection, bacteremia and infective endocarditis | Blood | Recurrent bacteremia on day 23 | Blood | Died |
| K303 | Positive | Pneumonia, bacteremia and spine infection (osteomyelitis, epidural abscess) | Blood | Recurrent bacteremia on day 64 | Blood | Survived |
| H045 | Negative | Nephrostomy tube related pyelonephritis and bacteremia | Blood | Recurrent pyelonephritis on day 37 | Urine from new nephrostomy tube | Survived |
| K081 | Negative | Line associated bacteremia | Blood | Recurrent bacteremia on day 49 | Blood | Survived |
| K186 | Negative | Septic arthritis with bacteremia and epidural abscess | Blood | Persistent bacteremia on day 10 | Blood | Survived |
| K213 | Negative | Spine osteomyelitis and bacteremia | Blood | Persistent spine osteomyelitis from bone culture on day 12 | Bone | Survived |
| N026 | Negative | Skin and soft tissue infection with deep abscess, osteomyelitis and bacteremia | Abscess  Blood | Recurrent deep abscess on day 44 | Abscess | Survived |
| N053 | Negative | Line associated bacteremia and septic thrombophlebitis | Blood | New pneumonia on day 68 | Bronchoscopy BAL culture | Survived |
| N068 | Negative | Skin and soft tissue infection with bacteremia | Blood | Recurrent bacteremia on day 55 | Blood | Survived |
| N088 | Negative | Prosthetic joint infection and bacteremia | Joint fluid  Blood | Recurrent prosthetic joint infection on day 52 | Joint fluid | Survived |
| K031 | Negative | Bacteremia and epidural abscess | Blood | New renal abscess on day 12 counted as persistent infection | Abscess | Survived |
| H029 | Negative | Skin and soft tissue infection with bacteremia | Blood | Recurrent bacteremia on day 41 | Blood | Survived |

Supplement Table 4. Sensitivity of outcomes being restricted to 30 days follow-up

|  | CzIE positive  (N=92)^a^ | CzIE negative  (N=167)^b^ | Unadjusted risk difference (95% CI) | Adjusted risk difference (95% CI) |
| --- | --- | --- | --- | --- |
| 30-day mortality | 14 (15.2%) | 25 (15.0%) | 0.2% (-8.4% to 10.2%) | 4.4% (-4.2% to 12.9%) |
| Microbiological treatment failure | 8 (8.7%) | 3 (1.8%) | 6.9% (1.7% to 14.6%) | 6.4% (0.8% to 11.9%) |

^a^For the 92 patients in the CzIE positive group at end of 30-days follow-up: 73 patients were alive without microbiological treatment failure, 5 patients had microbiological treatment failure but survived, 11 patients did not have microbiological treatment failure but died, 3 patients had microbiological treatment failure then died

^b^For the 167 patients in the CzIE negative group at end of 30 days follow-up: 139 patients were alive without microbiological treatment failure, 3 patients had microbiologic al treatment failure but survived, 25 patients did not have microbiologic al treatment failure but died, 0 patient had microbiologic al treatment failure then died

In a competing risk model, CzIE had an unadjusted sub-distribution hazard ratio (sHR) of 5.02 (95% CI 1.33 to 18.9) for microbiologic treatment failure. After overlap weighting of propensity scores, CzIE had an adjusted sHR of 4.43 (95% CI 1.03 to 19.1).

Supplement Table 5. Outcomes in subgroup analysis of patients with MSSA bacteremia

|  | CzIE positive  (N=73)^a^ | CzIE negative  (N=138)^b^ | Unadjusted risk difference (95% CI) | Adjusted risk difference (95% CI) |
| --- | --- | --- | --- | --- |
| 90-day mortality | 18 (24.7%) | 35 (25.4%) | -0.7% (-12.4% to 12.2%) | 0.9% (-10.9% to 12.6%) |
| Microbiological treatment failure | 13 (17.8%) | 10 (7.3%) | 10.6% (1.6% to 21.5%) | 7.6% (-1.9% to 17.1%) |

^a^For the 73 patients in the CzIE positive group at end of 90-days follow-up: 45 patients were alive without microbiological treatment failure, 10 patients had microbiological treatment failure but survived, 15 patients did not have microbiological treatment failure but died, 3 patients had microbiological treatment failure then died

^b^For the 138 patients in the CzIE negative group at end of 90 days follow-up: 93 patients were alive without microbiological treatment failure, 10 patients had microbiological treatment failure but survived, 35 patients did not have microbiological treatment failure but died, 0 patient had microbiological treatment failure then died

Supplement Table 6. Outcomes in subgroup analysis of patients with deep seated infection

|  | CzIE positive  (N=43)^a^ | CzIE negative  (N=78)^b^ | Unadjusted risk difference (95% CI) | Adjusted risk difference (95% CI) |
| --- | --- | --- | --- | --- |
| 90-day mortality | 8 (18.6%) | 12 (15.4%) | 3.2% (-10.1% to 18.9%) | 1.4% (-8.6% to 11.5%) |
| Microbiological treatment failure | 15 (34.9%) | 5 (6.4%) | 28.5% (14.2% to 44.2%) | 23.0% (8.4% to 37.9%) |

The following foci were considered to be deep seated infection with potentially high inoculum: infective endocarditis, vascular graft infection, septic arthritis, prosthetic joint infection, osteomyelitis, epidural abscess, other deep-seated abscess and empyema.

^a^For the 43 patients in the CzIE positive group at end of 90-days follow-up: 24 patients were alive without microbiological treatment failure, 11 patients had microbiological treatment failure but survived, 4 patients did not have microbiological treatment failure but died, 4 patients had microbiological treatment failure then died

^b^For the 78 patients in the CzIE negative group at end of 90 days follow-up: 61 patients were alive without microbiological treatment failure, 5 patients had microbiological treatment failure but survived, 12 patients did not have microbiological treatment failure but died, 0 patient had microbiological treatment failure then died

Supplement Table 7. Outcomes in subgroup of patients in whom source control is applicable stratified by whether source control was achieved

|  |  | CzIE positive  (N=57) | CzIE negative  (N=103) |
| --- | --- | --- | --- |
| 90-day mortality | Source control achieved | 4/47 (8.5%) | 12/89 (13.5%) |
|  | Source control not achieved | 4/10 (40.0%) | 7/14 (50.0%) |
| Microbiological treatment failure | Source control achieved | 14/47 (29.8%) | 8/89 (8.9%) |
|  | Source control not achieved | 2/10 (20.0%) | 1/14 (7.1%) |
